# Late-Onset Bilateral Choroidal Metastases from Clear Cell Renal Cell Carcinoma

**DOI:** 10.1155/2020/8862203

**Published:** 2020-12-09

**Authors:** Constantinos D. Georgakopoulos, Athina Pallikari, Panagiotis Plotas, Olga E. Makri

**Affiliations:** Department of Ophthalmology, Medical School, University of Patras, Rio, 265 04 Patras, Greece

## Abstract

**Aim:**

To present a case of clear cell renal cell carcinoma with late-onset bilateral choroidal metastases. *Case Report*. A 57-year-old male patient in the Oncology Clinic complained of reduced vision in the right eye (OD) for 7 days. The patient, who was under immunotherapy with nivolumab, had been diagnosed with clear cell renal cell carcinoma in the left kidney 15 years ago that recurred in the right kidney before 2 years. Metastases in the brain, lungs, and bones had also been diagnosed. On ophthalmological examination, the visual acuity was 20/50 OD and 20/20 in the left eye (OS). Dilated fundus examination in OD revealed a single raised oval-shaped yellowish choroidal nodule infratemporally with macular involvement. A similar lesion, sparing the macula, was observed in OS. Fundus autofluorescence revealed diffuse punctate hyperautofluorescence on the lesions. Serous macular detachment was also observed in OD. A standardized A-scan ultrasound demonstrated an irregular structure of the lesions with moderate to high internal reflectivity. Based on the history and clinical and echographic characteristics, the diagnosis of bilateral choroidal metastases from renal cell carcinoma was set.

**Conclusion:**

Choroidal metastases from the primary renal tumor are extremely rare. The time interval between primary malignancy and choroidal metastasis is reported to be 12-96 months. Bilateral choroidal metastases have been described in 9 cases. We describe a rare case where bilateral choroidal metastases were diagnosed 15 years after the initial diagnosis of clear cell renal cell carcinoma.

## 1. Introduction

Renal cell carcinoma (RCC) is a rare but potentially fatal malignancy among adults, as it is characterized by a strong tendency to metastasize unpredictably to any organ, even decades after surgical removal of the primary tumor [1]. Approximately one-third of patients with RCC will eventually develop a distant metastatic tumor, a fact that results in a guarded prognosis [2]. The most common metastatic locations are the lungs, bones, liver, and brain, whereas the uveal tract is an extremely rare site of kidney tumor spread as it represents about 4% of all uveal metastatic malignancies [3].

The choroid is one of the most highly vascularized tissues of the body and comprises a common site of uveal metastatic disease. A large retrospective chart review reported choroidal involvement in 88% of the 950 cases with uveal metastases studied [4]. Breast and lung cancer are the most common primary tumor sites in cases of choroidal metastases, while metastases originating from kidneys are rare [3, 4].

We present a case of bilateral choroidal metastases originating from RCC that occurred 15 years after the diagnosis of the primary tumor.

## 2. Case Presentation

A 57-year-old hospitalized male patient presented with decreased vision in the right eye (OD) during the last week. His medical history was significant with left radical nephrectomy before 15 years due to a histologically proven clear cell renal carcinoma (Fuhrman grade II [5]). Tumor recurrence was detected 2 years ago, and the patient underwent right heminephrectomy (stage pT1bNx according to the 8^th^ edition of the AJCC Cancer Staging Form). Secondary brain, pulmonary, and bone metastases had also been developed. The patient was under immunotherapy with nivolumab.

**Figure 1:**
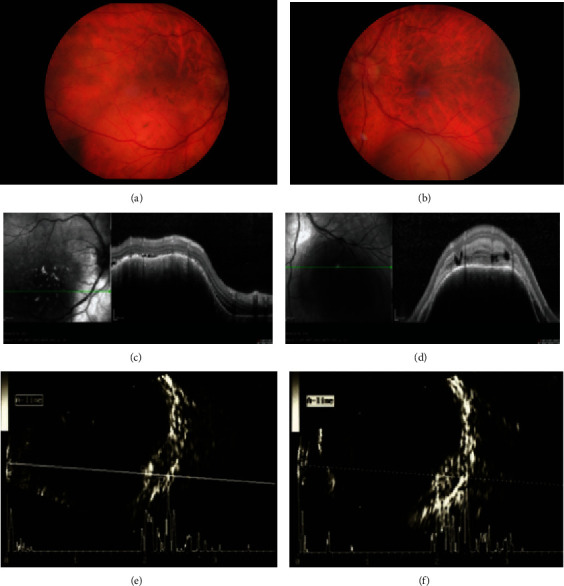
Color fundus image of the right eye (a) revealed an elevated, oval-shaped, lightly pigmented, orange, subretinal lesion in the inferotemporal quadrant with secondary serous retinal detachment extending to the macula. In the left eye (b), a nonpigmented, reddish-white, subretinal lesion below the inferior temporal arcade was observed. Examination with optical coherence tomography in the right eye (c) revealed eradication of the choriocapillaries and irregular choroidal surface overlying the lesion and in the left eye (d) showed a dome-shaped choroidal morphology, compression of the overlying choriocapillaris, and intraretinal fluid at the site of the lesion. Ultrasound examination showed dome-shaped choroidal lesions in the right (e) and left (f) eyes with a medium-to-high internal reflectivity and a dense acoustic sign.

On ophthalmologic examination, his best-corrected visual acuity was 20/50 in (OD) and 20/20 in the left eye (OS). The slit-lamp biomicroscopy of the anterior segment was unremarkable in both eyes. Funduscopic examination of OD showed an elevated, oval-shaped, lightly pigmented, orange, subretinal lesion at the inferotemporal quadrant with secondary serous retinal detachment that extended to the macula (Figure 1(a)). A flat pigmented naevus temporal to the lesion was also present. The fundoscopy of OS revealed a nonpigmented, reddish-white, subretinal lesion below the inferior temporal arcade (Figure 1(b)).

Optical coherence tomography (OCT) in OD demonstrated eradication of the choriocapillaries and irregular choroidal surface overlying the lesion associated with a serous retinal detachment that extended to the macula (Figure 1(c)). In OS, OCT showed a dome-shaped choroidal morphology, compression of the overlying choriocapillaries, and intraretinal fluid at the site of the lesion (Figure 1(d)). Fundus autofluorescence revealed scattered hyperfluorescent clumps of the lesion in OD, whereas the lesion in OS was hypofluorescent. Fluorescence angiography was contraindicated.

Ultrasound examination showed bilateral dome-shaped choroidal lesions measuring 8.2 mm base and 3.0 mm height in OD and 7.2 mm base and 3.4 mm height in OS (Figure 1 (e), (f)). The lesions had medium-to-high internal reflectivity with a dense acoustic sign.

Based on the medical history and the clinical and multimodal imaging findings, the patient was diagnosed with bilateral choroidal metastases originating from a RCC that occurred 15 years ago. Due to the patient's low life expectancy and poor systemic status, it was decided not to proceed to biopsy of the metastatic sites and any local therapeutic regimen while systemic immunotherapy was continued. The patient expired 3 months after the occurrence of choroidal metastases.

## 3. Discussion

Ocular metastases comprise an extremely uncommon site of distant spread from RCC and may be the initial manifestation of a previously undiagnosed RCC. The uveal tract is the most frequent site of ocular metastatic disease from RCC [6] (Table 1). Haimovici et al. presented 5 cases with RCC-related choroidal metastases that occurred 6-18 years after nephrectomy [7]. In a retrospective review, Sountoulides et al. documented 19 cases with RCC suffered from ocular metastatic disease. Interestingly, the choroid was implicated in 4 out of 19 cases, and the time interval between the diagnosis of RCC and metastasis varied from 1 to 8 years [6]. There is one rare case of unilateral choroidal metastasis that developed 25 years after nephrectomy due to RCC [8].

A review of the literature revealed a small number of cases with bilateral kidney-related choroidal metastases. More precisely, in a retrospective review of 1111 patients with uveal metastases, bilateral kidney cancer-related choroidal metastases were found in 7 out of 46 cases with metastatic kidney tumor [3]. The orangish appearance of the uveal mass may indicate renal origin, but may also be found in metastases originating from a thyroid carcinoma or a carcinoid tumor [7]. Additionally, RCC choroidal metastases are usually characterized by a slightly higher height-to-base ratio in A-scan ultrasonography compared to those originating from other metastatic cancers, whilst the mean thickness of the mass has been reported to be approximately 4 mm [4].

The diagnosis of a choroidal metastasis is based on medical history and ophthalmoscopic and echographic characteristics, whereas uncertain cases require fine needle aspiration biopsy [4, 7]. Differential diagnosis of choroidal metastases includes choroidal melanomas, haemangiomas, granulomas, and choroidal osteomas [9]. The treatment depends on the systemic status of the patient and the number and location of choroidal tumors. In cases with poor systemic status, observation is preferred. Other treatment modalities include systemic chemotherapy with drugs used for the primary cancer, immunotherapy, hormone therapy, or radiotherapy in multifocal and bilateral metastases. Proton beam radiotherapy, plaque radiotherapy, transpupillary radiotherapy, or photodynamic therapy is the preferred treatment strategy for solitary metastasis while enucleation is suggested for blind painful eyes [9].

In conclusion, we describe an extremely rare case where bilateral choroidal metastases were diagnosed 15 years after the initial diagnosis of a clear cell RCC. Choroidal metastases from RCC represent a rare condition that should not be overlooked. Therefore, a high index of suspicion is required with sufficient investigation of patients with a history of RCC who complain of visual symptoms, even decades after the RCC diagnosis.

## Figures and Tables

**Table 1:** Cases of renal cell carcinoma with metastases to the choroid. In nine cases, the choroidal metastases were bilateral.

Study	Number of cases	Mean age at diagnosis (years)	Sex		Laterality		Diagnosis of metastasis before primary tumor	Interval between diagnosis of primary tumor and metastasis (years)
			Male	Female	Unilateral	Bilateral		
Shields et al. [3]	46	66	34	12	39	7	2	N/A
Sountoulides et al. [6]	4	49.6	3		N/A	N/A	0	1-8
Haimovici et al. [7]	5	61.4	4	1	4	1	2	6-18
Bellerive et al. [8]	1	73	1			1	0	25

## Data Availability

The authors confirm that the data supporting the findings of this study are available within the article.
